# Bones, Glands, Ears and More: The Multiple Roles of FGF10 in Craniofacial Development

**DOI:** 10.3389/fgene.2018.00542

**Published:** 2018-11-16

**Authors:** Michaela Prochazkova, Jan Prochazka, Pauline Marangoni, Ophir D. Klein

**Affiliations:** ^1^Laboratory of Transgenic Models of Diseases, Czech Centre for Phenogenomics, Institute of Molecular Genetics, Czech Academy of Sciences, Prague, Czechia; ^2^Program in Craniofacial Biology, Departments of Orofacial Sciences and Pediatrics, Institute for Human Genetics, University of California, San Francisco, San Francisco, CA, United States

**Keywords:** FGF10, craniofacial development, palate, salivary gland, lacrimal gland, inner ear, eyelid, taste papillae

## Abstract

Members of the fibroblast growth factor (FGF) family have myriad functions during development of both non-vertebrate and vertebrate organisms. One of these family members, *FGF10*, is largely expressed in mesenchymal tissues and is essential for postnatal life because of its critical role in development of the craniofacial complex, as well as in lung branching. Here, we review the function of FGF10 in morphogenesis of craniofacial organs. Genetic mouse models have demonstrated that the dysregulation or absence of FGF10 function affects the process of palate closure, and FGF10 is also required for development of salivary and lacrimal glands, the inner ear, eye lids, tongue taste papillae, teeth, and skull bones. Importantly, mutations within the *FGF10* locus have been described in connection with craniofacial malformations in humans. A detailed understanding of craniofacial defects caused by dysregulation of FGF10 and the precise mechanisms that underlie them offers new opportunities for development of medical treatments for patients with birth defects and for regenerative approaches for cancer patients with damaged gland tissues.

## Introduction

FGF10 is a member of the fibroblast growth factor (FGF) family, a highly evolutionarily conserved group of proteins that trigger signaling via receptor tyrosine kinases. The FGF signaling pathway plays central roles in developmental processes from head to toe, including formation of the brain, limbs, kidneys, hair follicles, and body axis elongation (Rosenquist and Martin, 1996; Lewandoski et al., 2000; Basson et al., 2008; Walker et al., 2016; Oginuma et al., 2017). The FGF family contains 22 ligands grouped into 7 subfamilies, and these ligands can bind to 4 receptors (FGFR1–4) (Ornitz and Itoh, 2001). The interaction of FGF ligands with their receptors is regulated by the extracellular environment, through proteoglycan cofactors and extracellular binding proteins. Activation of FGF receptors involves phosphorylation of specific tyrosine residues that mediate interaction with cytosolic adaptor proteins and the RAS-MAPK, PI3K-AKT, PLCγ, and STAT intracellular signaling pathways (Ornitz and Itoh, 2015). FGF10 is a canonical FGF and belongs to the FGF7 subfamily, together with FGF3, FGF7, and FGF22. The common feature of these FGF ligands is their specific binding of the IIIb splice variant of FGFR 1 and 2 (Zhang et al., 2006). Moreover, during organogenesis FGF10 serves as a major ligand for the FGFR 2 IIIb isoform, which localizes to the epithelium (Ohuchi et al., 2000), and in general *Fgf10* is predominantly expressed in the mesenchyme, with the protein it encodes signaling to the epithelium.

**FIGURE 1:**
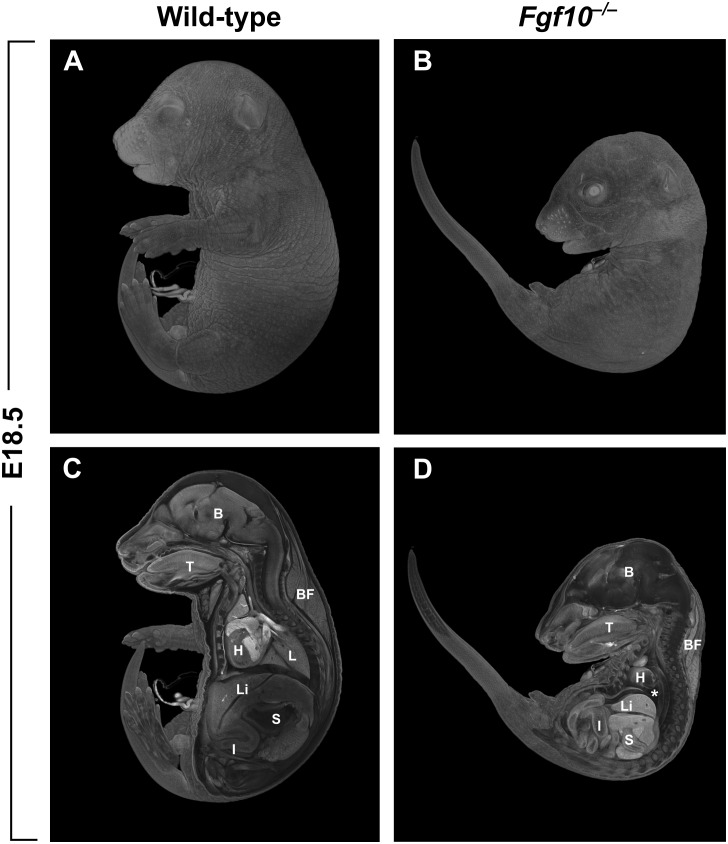
**(A,B)** Whole-mount μCT images of wild-type and *Fgf10* null mouse embryos at E18.5. **(C,D)** Medial cross-sections through wild-type and mutant embryo. *Fgf10* mutant embryos show complete amelia; another pronounced phenotype is lung agenesis (asterisk). B, brain; BF, brown fat; H, heart; I, intestines; L, lungs; Li, liver; S, stomach; T, tongue.

The majority of studies on the role of FGF10 in vertebrates have been performed using mice carrying null mutations in *Fgf10*. In addition to the craniofacial complex, many other organs of the body are affected in the *Fgf10* null mutants. Among the most prominent phenotypes in the mutants are that both hindlimbs and forelimbs are completely missing (amelia), and there is lung agenesis (Ohuchi et al., 2000; Figure 1). Perinatal lethality in the *Fgf10* mutants results from respiratory failure. Notably, the phenotype of *Fgfr2* mutant mice almost completely overlaps with that of *Fgf10* mutants (Ohuchi et al., 2000).

Mutations in *FGF10* have been found to cause numerous developmental defects and pathologies in humans. For example, loss-of-function mutations in *FGF10* have been reported to cause LADD (Lacrimo-auriculo-dento-digital) syndrome (Milunsky et al., 2006; Shams et al., 2007), which affects multiple organs, the majority of which are in the craniofacial complex. This and other human conditions connected to craniofacial development are further discussed below.

## Role of FGF10 in Craniofacial Morphogenesis

*Fgf10* is expressed largely in the mesenchyme of many developing structures within the craniofacial complex, including teeth, tongue and palatal shelves, and it signals to epithelia where *Fgfr2* is expressed. Mutations in *Fgf10* lead to a wide range of defects, emphasizing the central importance of FGF10 signaling in many developmental processes.

### Palatogenesis

**FIGURE 2:**
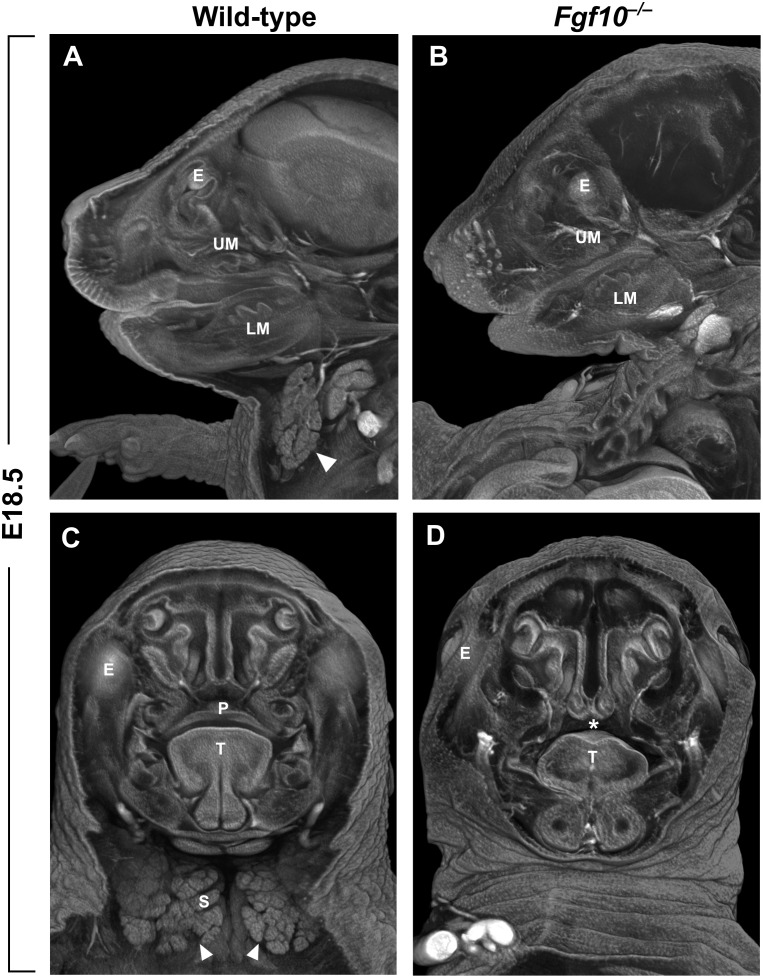
**(A–D)** Sagittal **(A,B)** and frontal **(C,D)** μCT sections of wild-type and *Fgf10* null embryos at E18.5. Absence of submandibular salivary gland (structure marked by arrowhead in wild-type in panels **A,C**) and cleft palate phenotype (asterisk in panel **D**) can be observed in *Fgf10* null embryos. E, eye; LM, lower molar; P, palate; T, tongue; S, salivary gland; UM, upper molar.

FGF10 is crucial for the process of closure of the secondary palate. Both *Fgf10* (Figures 2C,D) and *Fgfr2* null mouse strains exhibit cleft palate with complete penetrance (Rice et al., 2004). *Fgf10* is expressed most strongly between embryonic day (E)11 and E13 in the mesenchyme of the anterior and middle portion of the shelves (Rice et al., 2004; Alappat et al., 2005). During this developmental period, palatal shelf outgrowth occurs prior to the subsequent elevation and fusion of the shelves between E14 and E15. At later stages, the *Fgf10* mutant shelves are shorter, square in shape, and missing the finger-like projections that normally reach each other and fuse (Rice et al., 2004). This change in morphology can be explained by differences in the regulation of cell proliferation and apoptosis. While one study reported that there are no apparent differences in the overall proliferation of the shelves (Alappat et al., 2005), another suggested that proliferation of epithelial cells is decreased in the FGF10 deficient palatal shelves along with downregulation of the morphogen encoded by *Shh* (Rice et al., 2004). Both studies then showed a significant increase in apoptosis mainly in the medial edge epithelium of the developing shelves (Rice et al., 2004; Alappat et al., 2005). Despite the discrepancies, it appears that FGF10 signals from the palatal mesenchyme to the epithelium and affects the cell fate and subsequently the outgrowth and shape of the palatal shelves.

Besides cell proliferation and survival, another mechanism possibly contributing to the formation of cleft palate in *Fgf10* mutants is the presence of aberrant adhesions of the epithelium of the shelves with the epithelium of the tongue or with other parts of the oral epithelium (Rice et al., 2004; Alappat et al., 2005). Presence of these fusions likely prevents the horizontalization (elevation) process of the palatal shelves, so they are kept in a vertical position and cannot begin to reach each other. Of note, when the tissue explants of the palatal shelves of *Fgfr2*^-/-^ mice were isolated and cultured *in vitro* in close proximity, the epithelia fused normally (Rice et al., 2004). The molecular basis behind the tendency to form aberrant epithelial fusions may be related to the regulation of Notch signaling by FGF10. Mutations in the Notch ligand *Jagged2* cause cleft palate with unelevated shelves heavily fused to the tongue epithelium (Jiang et al., 1998), and the *Fgf10* mutants exhibit severe downregulation of *Jagged2* expression within the palatal shelf epithelium at E12.5 (Alappat et al., 2005). This suggests that FGF10 is upstream of Notch signaling in the developing palatal shelves and affects the ability and correct timing of their fusion potential.

Tongue morphology is also altered in the *Fgf10* mutants. Likely due to the presence of aberrant epithelial fusions, the tongue does not descend as it should, which perturbs this necessary step in the process of shelf elevation (Rice et al., 2004). Indeed, a partial ankylosis of the tongue (adherence to the floor of mouth accompanied by immobility) is present in the *Fgf10* mutant embryos (Rice et al., 2004). Notably, overexpression of *Fgf10* also affects the tongue shape and can lead to cleft palate. This phenomenon was described in mice with neural crest-specific *Tak1* deletion, which affects TGFβ signaling, in turn leading to activation of FGF10, higher cell proliferation, and significantly increased height of the tongue that prevents the elevation of palatal shelves (Song et al., 2013). The role of TGFβ signaling upstream of FGF10 in morphogenesis of the tongue was also confirmed when *Tgfbr2* was conditionally deleted in neural crest cell progeny, as the addition of endogenous FGF10 rescued the muscle cell number in mutant tongues (Hosokawa et al., 2010). FGF10 also regulates tongue taste papillae development, which is discussed below.

In humans, genome-wide association studies (GWAS) have shown that SNPs near *FGF10* are highly associated with cleft lip and/or palate (Shi et al., 2009; Yu et al., 2017). Likely, due to their different orofacial shape with a more prominent rostral component, cleft lip does not typically occur spontaneously in mice, and it is rarely observed even with genetic or environmental challenge. Therefore, this model organism is theoretically not an ideal one to study cleft lip etiology. Nevertheless, there are certain mouse strains that are susceptible to developing cleft lip, e.g., the group of so-called A strains that exhibit smaller midface size compared to other strains (Young et al., 2007). Among the A strains, A/WySn has the highest spontaneous incidence of cleft lip, ranging between 20 and 30% (Juriloff, 1982). The high prevalence and susceptibility of these mice to cleft lip is thought to be caused by a mutation in *Wnt9b*, which is also on the list of top clefting genes from human GWAS data (Juriloff et al., 2006; Yu et al., 2017) *Wnt9b* knockout mice exhibit cleft lip and, importantly, the expression of *Fgf8*, *Fgf10*, and *Fgf17* is down-regulated in the tissue of facial processes forming the future lip in these mice. Taken together, the data from GWAS along with the data from susceptible mouse strains suggest a role for FGF10 in lip development, despite the absence of cleft lip in *Fgf10*^-/-^ mice.

Notably, soft palate development is also dependent on FGF10, and this cannot be evaluated in *Fgf10* null mutants, because the wide hard cleft palate interferes with the later development of the soft palate. Loss of *Dlx5* leads to shortening of the soft palate and absence of adjacent muscles that are derived from the fourth pharyngeal arch. *Fgf10* was shown to lie downstream of DLX5, and the *Dlx5* mutant phenotype can be rescued by addition of FGF10 (Sugii et al., 2017).

### Eye Lid Development

**FIGURE 3:**
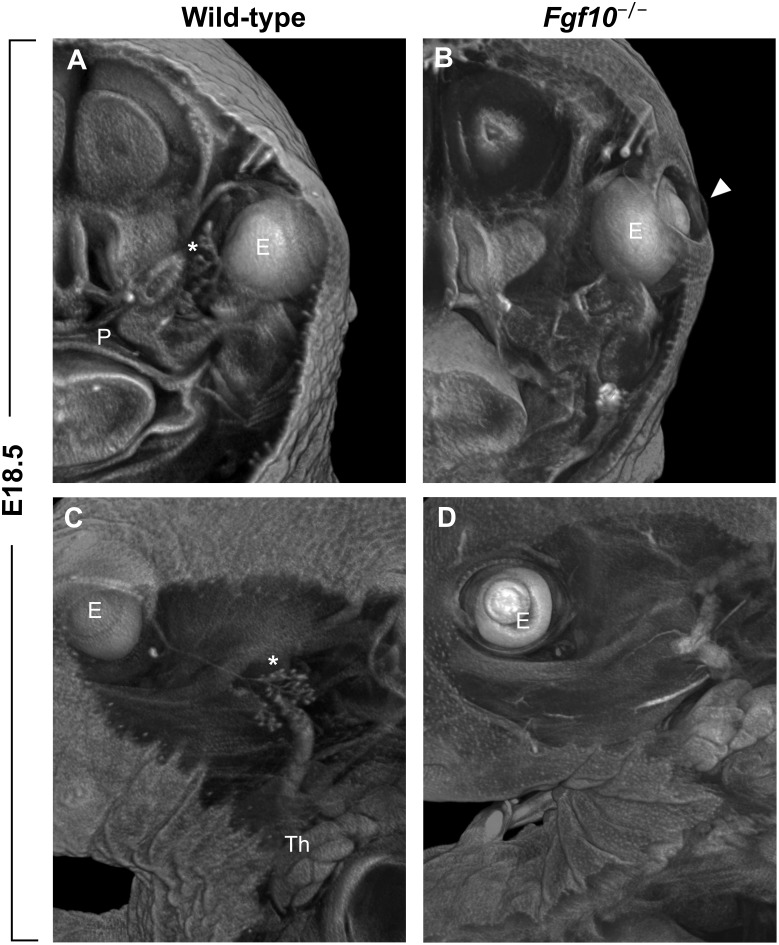
**(A–D)** Frontal **(A,B)** and sagittal **(C,D)** μCT sections of wild-type and *Fgf10* null embryos at E18.5. Note absence of eyelid (arrowhead in panel **B**) and hypoplasia of ocular glands (Harderian and extraorbital lacrimal glands marked by asterisks in panels **A,C**, respectively) in *Fgf10* null embryos. E, eye; P, palate; Th, thymus.

Another clefting-like pathology in the craniofacial area is the phenotype of open eyelids in *Fgf10* null mice at prenatal stages when the eye is normally covered by skin (Figures 1B, 3B). The absence of *Fgf10*, which is normally expressed in the mesenchyme beneath the protruding epidermal cells of the nascent eyelid, causes a decrease in proliferation of these cells as well as changes in their shape, along with hampering their coordinated migration (Tao et al., 2005). These effects are due to downstream regulation of pathways important for these processes, including activin, TGFα, and SHH (Tao et al., 2005).

### Skull Morphology

A major group of human craniosynostosis syndromes, including Apert, Crouzon, or Pfeiffer syndromes, is caused by mutations leading to overactivation of Fgf receptors. Apert and Crouzon syndromes are caused by mutations in *FGFR2* that increase affinity of the receptor for the ligand, and Pfeiffer syndrome is caused by mutations in either *FGFR2* or *FGFR1* (Schell et al., 1995; Anderson et al., 1998; Hibberd et al., 2016). The search for specific ligands involved in the process of fusion of the sutures revealed that FGF10 can play a significant role in proper formation of skull shape. *Fgf10* mRNA is present in the osteoprogenitors in the frontal bone condensation (Veistinen et al., 2009), and genetic knock-down of *Fgf10* rescues the skeletal phenotype in an Apert syndrome mouse model *FgfR2*-IIIc^+/Δ^ (Hajihosseini et al., 2009). When compared perinatally, the *Fgf10* null embryos do not exhibit pathological morphology of calvarial bones, while the *FgfR2*-IIIc^+/Δ^ mice already lack the coronal suture (Hajihosseini et al., 2009). Unfortunately, postnatal development of skull bones and sutures cannot be followed in the perinatal lethal *Fgf10* null mutants, so it is not possible to exclude that the loss of FGF10 has an impact on skull morphology. Nevertheless, it is instructive to consider the Apert syndrome model, in which the mice are hemizygous for *Fgfr2 IIIC* and exhibit a splicing switch resulting in ectopic expression of *FGFR2 IIIb* in calvarial mesenchyme; similar mutations are only rarely found in humans (Hajihosseini et al., 2001, 2009; Bochukova et al., 2009). More than 98% of Apert syndrome patients carry either Ser252Trp or Pro253Arg missense gain-of-function mutations in the IIIa exon (common for IIIb and IIIc variants). These mutations likely predominantly cause the skull defects through aberrant function of FGFR2 IIIc, which is involved in proper bone formation (Eswarakumar et al., 2002). Taken together, these findings suggest that FGF10 may be dispensable for the properly timed fusion of sutures and skull development, but unphysiologically high and/or ectopically activated FGFR2 signaling triggered by FGF10 can cause developmental defects of these structures.

### Sensory Organs

FGF10 also affects the development of organs that possess a sensory function or will sustain it postnatally, including, as mentioned above, the taste papillae of the tongue. The mammalian tongue epithelium contains three types of papillary structures that house taste cells – the foliate, fungiform, and circumvallate (CVP) papillae. The multiple fungiform papillae covering approximately two thirds of the tongue dorsum and the posteriorly situated CVP have been shown to be regulated by FGF10 (Petersen et al., 2011; Prochazkova et al., 2017). Interestingly, the effect of *Fgf10* is opposite in these two types of taste papillae. The CVP, which is normally a single structure in mouse, is absent or diminished in *Fgf10* null murine tongues, whereas the overactivation of RTK signaling in embryos carrying mutations in the RTK negative feedback regulator *Sprouty (Spry)* genes led to enlargement of the papillary field and presence of multiple CVPs (Petersen et al., 2011). In contrast, the development of fungiform papillae is negatively affected by the level of FGF10, such that the fungiform papillae of *Fgf10*^-/-^ tongues are significantly larger, and in *Spry2*^-/-^ tongues with increased FGF signaling they are much smaller. Notably, fungiform size is controlled by FGF10, but the overall patterning is not; at a mechanistic level, the downstream action of FGF10 is likely exerted by affecting the diffusion as opposed to the transcription of Wnt ligands (Prochazkova et al., 2017). The difference in regulation of papillary area in CVP and fungiform papillae might result from a different developmental origin of the part of the tongue covered by fungiform papillae (ectodermal) versus the posterior part near the root of the tongue housing the CVP (endodermal) (Rothova et al., 2012). Whether the level of FGF10 signaling can impact the quality of taste remains an open question.

Another sensory organ with dysregulated development in *Fgf10* mutant embryos is the inner ear. Absence of FGF10 leads to complete agenesis of the posterior semicircular canal. In addition, malformations are present in the anterior and lateral canals as well as in the positioning of the remaining sensory epithelia with respect to the utricle; defects were also observed in the cilia of hair cells (Pauley et al., 2003). Interestingly, heterozygous *Fgf10*^+/-^ mice also exhibit reduction or even absence of the posterior canal, suggesting a strong dependence on FGF10 dosage during development of this structure (Urness et al., 2015). In addition to the motion detection part of the inner ear, the *Fgf10* mutant embryos also exhibit pathologies in morphology of cochlear non-sensory regions, including shorter and narrower duct, absence of Reissner’s membrane within the cochlear epithelium, and agenesis of a large portion of the outer sulcus (Urness et al., 2015). Even though these structures belong to the non-sensory part of the cochlea, both the Reissner’s membrane and the outer sulcus are important for maintenance of the endolymph homeostasis and therefore necessary for hearing. Similar defects might be present also in humans and explain a part of the phenotype LADD syndrome caused by mutations in *FGF10 or FGFR2* (Milunsky et al., 2006). More than half of the affected individuals suffer from hearing loss, and cochlear hypoplasia was also observed in some of the patients (Lemmerling et al., 1999; Milunsky et al., 2006). The severity of hearing defects might be more pronounced when a causative mutation in FGFR2 is present, as FGF10 has a redundant role with FGF3 during inner ear formation – the murine double mutants for these FGFs fail to form otic vesicles (Alvarez et al., 2003; Wright, 2003). Notably, the FGFR2 IIIb knock-out mice exhibit more severe phenotypes than single *Fgf3* and *Fgf10* mutants, but their inner ear is affected less than in the *Fgf3/Fgf10* dKOs (Pirvola et al., 2000; Alvarez et al., 2003). This discrepancy suggests that FGF3 and FGF10 in the ear region can possibly also bind other FGF receptors, such as FGFR1, which has affinity for these two ligands (Zhang et al., 2006). The FGFR2 IIIc form may also be activated by FGF3/10, because the general FGFR2 mutant has a more pronounced phenotype than FGFR2 IIIb only (Xu et al., 1998; Alvarez et al., 2003). However, this might be explained by an additional role of FGF8 during early inner ear development (Domínguez-Frutos et al., 2009).

*Fgf10* is also highly expressed in the external ear (pinna) of mouse embryos (El Agha et al., 2012) and, interestingly, one of the defects observed in the LADD patients are low-set, cup-shaped ears. Nevertheless, no external ear abnormalities have so far been described in direct connection to FGF10 (see normal pinna in *Fgf10* mutant embryos in Figure 1). It may be again a case of compensation by another FGF ligand and their common dysfunction in patients with *FGFR2* rather than *FGF10* mutations *per se*.

### Development of Teeth and Mandible

The molar tooth germ is a widely used model for studying epithelial morphogenesis and epithelial–mesenchymal interactions. In mouse, tooth development starts at ∼E11.5 with active rearrangement of epithelial cells in the posterior area of the jaws, where FGF8 serves as a major signaling molecule (Prochazka et al., 2015). A cylindrical epithelial invagination called the dental lamina is formed at E12.5, and at E13.5, progressive budding of epithelium from the dental lamina takes place, which is supported by condensing neural crest-derived cells expressing *Fgf3* and *Fgf10* (Kettunen et al., 2000). The rapid epithelial ingrowth is accompanied by formation of a signaling center called the enamel knot. Mesenchymal *Fgf10* is expressed in the area of the mandible where future molar teeth form, and complete agenesis of molars was described in *Fgfr2* deficient mouse embryos, but with the loss of *Fgf10* only minor morphological defects are observed in molar development (Ohuchi et al., 2000); the absence of a dramatic *Fgf10* mutant tooth phenotype is likely due to compensation by *Fgf3*. The budding process of the molar primordia in *Fgf10*^-/-^ embryos is delayed around E13, but at later stages tooth development catches up, and the final molar tooth is only slightly smaller in size compared to wild-type (Ohuchi et al., 2000; Veistinen et al., 2009; Figure 2).

In rodents, the incisors are evergrowing, with a population of adult stem cells present in the most proximal region called the cervical loop (Harada et al., 1999). *Fgf10* plays a major role in maintenance of the stem cell niche of the mouse incisor by regulation of Notch signaling in the dental epithelium (Harada et al., 1999). The *Fgf10* null embryo incisor is apparently smaller, mainly because of an absent cervical loop (Ohuchi et al., 2000; Harada et al., 2002). Related to this, FGF10 has been suggested as a principal morphogenetic factor driving the teeth toward an evergrowing fate, as *Fgf10* expression is maintained in continuously growing teeth (e.g., mouse incisor or vole molar) throughout life, and the *Fgf10* mutant incisors lose continuously growing features when cultured in kidney capsules (Yokohama-Tamaki et al., 2006).

One group of pathologies associated with LADD syndrome are dental defects. The patients often have underdeveloped teeth with thin enamel and peg-shaped incisors. Even though molar development does not seem to be severely affected in the absence of FGF10 when evaluated prenatally in the mouse model, the findings in LADD patients support the role of FGF10 in tooth development. Some of the LADD patients suffering from dental pathologies may carry a specific genetic alteration in *FGFR2* (Rohmann et al., 2006; Shams et al., 2007). However, there are also reports of patients with enamel hypoplasia or small teeth with disrupted caps and crown morphology that are associated with *FGF10* mutations (Milunsky et al., 2006). Moreover, increased expression of *FGF10* along with *FGF7* was found in samples from human ameloblastoma, a benign jaw tumor originating from the cells of odontogenic epithelium, and FGF10 was shown to directly support proliferation of these cells (Nakao et al., 2013). The mild phenotype and normal cell-differentiation gradient of ameloblasts and odontoblasts in *Fgf10* null embryos (Harada et al., 2002) suggest that human dental development might differ from that of mice. Because the post-eruption dentition cannot be studied in the perinatal lethal *Fgf10*^-/-^ mice, conditional models will be needed in the future.

Similarly to tooth development, mandibular morphogenesis is not severely altered in *Fgf10* null embryos. Nevertheless, the developing jaw is apparently sensitive to the dosage of FGF10, as in the rat model, *Fgf10* overexpression was described to cause elongation of Meckel’s cartilage and enhanced chondrogenic differentiation within the mandible. Notably, proliferation of mandibular cells was not affected by higher levels of FGF10, and the longer Meckel’s cartilage was deformed and spiral-shaped, which affected the final shape of the jaw (Terao et al., 2011). The importance of FGF10 for proper mandibular development is also supported by association between genetic polymorphisms in *FGF10* and mandibular prognathism in humans (Cruz et al., 2017).

### Salivary and Lacrimal Glands

As with its critical role during lung development, FGF10 plays an important role in morphogenesis of branching organs within the craniofacial complex, including the salivary and lacrimal glands. The expression of *Fgf10* is high in the mesenchyme surrounding the developing salivary glands. *Fgf10* null embryos display aplasia of the salivary glands (Figure 2) with their development arrested at the bud stage (Ohuchi et al., 2000; Jaskoll et al., 2005). FGF10 acts upstream of SOX9 to positively regulate the progenitor cell population and drive outgrowth of the glands (Chatzeli et al., 2017). Furthermore, explant cultures of salivary gland tissue can recapitulate the physiological branching morphogenesis *in vitro* only if the epithelium is cultured with the surrounding mesenchyme or if FGF10 is added to the culture of the isolated epithelial tissue (Rebustini and Hoffman, 2009; Knosp et al., 2012). Notably, regulation of binding affinity of FGF10 to heparan sulfate is a decisive feature in the balance between promoting gland morphogenesis fate toward branching versus elongation (Patel et al., 2007; Makarenkova et al., 2009). FGF10 dose-dependence during development of salivary glands is further supported by the fact that mice heterozygous for *Fgf10* have hypoplastic salivary glands and xerostomia (dry mouth) (Jaskoll et al., 2005; May et al., 2015).

The role of FGF10 in lacrimal gland development is similar to its role in salivary gland morphogenesis. *Fgf10*, which is expressed in the mesenchyme adjacent to developing lacrimal epithelial bud, induces lacrimal gland development, and *Fgf10* null murine embryos exhibit agenesis of all ocular glands – the extraorbital and intraorbital lacrimal glands as well as the Harderian gland (Govindarajan et al., 2000; Makarenkova et al., 2000; Figure 3). The proteoglycans at the cell surface and in the extracellular matrix also affect lacrimal gland morphogenesis – the *O*-sulfation of heparan sulfate was shown to be essential for FGF10–FGFR2 interaction on lacrimal gland cell surface (Qu et al., 2011). In addition to the large orofacial glands, FGF10 also plays an important role during development of nasal submucosal glands responsible for mucus secretion in airways (May et al., 2016).

Patients with ALSG (aplasia of the lacrimal and major salivary glands) exhibit both salivary and lacrimal phenotypes, and this rare disorder is caused by loss-of-function mutations in *FGF10* (Entesarian et al., 2007; Scheckenbach et al., 2008; Seymen et al., 2017). ALSG patients suffer from xerostomia and dental decay, eye irritation, and epiphora (excessive tearing). In contrast, LADD syndrome covers a wider spectrum of malformations, including the above mentioned dental and auditory defects and also an abnormal number of fingers or digits. Nevertheless, LADD syndrome overlaps with ALSG in terms of lacrimal and salivary defects, and thus these two autosomal dominant disorders are considered part of the same phenotypic spectrum. The data from affected families support this idea, with reports of a daughter with typical features of LADD inheriting the mutation from her mother with ALSG (Milunsky et al., 2006). Taken together, the human clinical data confirm the importance of the correct function and level of FGF10 in the development of craniofacial structures, even though the precise regulation and severity of the phenotype apparently depend on both genetic and environmental factors. A systematically generated overview of the phenotypes in *Fgf10* null embryos is available at the International Mouse Phenotype Consortium (IMPC) database: www.mousephenotype.org.

## Summary and Discussion

FGF10 signaling plays important roles in the development of many craniofacial structures. FGF10 is required for the branching morphogenesis of salivary and lacrimal glands, for the closure of the secondary palate, and for eyelid development; it also affects the structure of the inner ear, taste papillae on the tongue, and the shape of the teeth and skull. The craniofacial phenotypes connected to FGF10 function along with known expression data are summarized in Supplementary Table 1.

FGF10 is predominantly expressed in the mesenchyme of developing structures and signals to adjacent epithelium. In contrast to this classical epithelial–mesenchymal interaction, structures of the inner ear exhibit strong epithelial expression of both *Fgf10* and *Fgfr2 IIIb* during development suggesting dependence on paracrine signaling (Pirvola et al., 2000; Pauley et al., 2003). Epithelial expression of *Fgf10* within orofacial tissues was described also in early oral epithelium (Kettunen et al., 2000). Nevertheless, the conditional *Fgf10* knock-out in neural crest cells using Wnt1-Cre phenocopied the tooth as well as oral cavity glands’ phenotype of the full knock-out and generally confirmed that, in orofacial structures comprised of mesenchyme originated fully from neural crest, the mesenchymal FGF10 plays the major role (Teshima et al., 2016).

FGF10 exerts its function in development via diverse and complex mechanisms. Perhaps the most widespread of these is a direct or indirect influence on epithelial cell proliferation and apoptosis, as in eyelid (Tao et al., 2005) or palate development (Rice et al., 2004). Nevertheless, multiple other actions of FGF10, such as regulation of migration or effect on adhesive behavior of the oral epithelium, have also been described in these organs. Control of proper morphogenesis and cell differentiation has also been proposed as one of the roles of FGF10 in many organs, such as salivary gland or inner ear (Alvarez et al., 2003; Makarenkova et al., 2009).

The striking overlap between phenotypes of *Fgf10* and *Fgfr2* null mice explains why FGF10 is considered as the major ligand of FGFR2 IIIb. Multiple FGFs can activate both FGFR2 IIIb (FGF3, 7, and 22 from the Fgf7 subfamily; but also FGF1) and FGFR2 IIIc (e.g., FGF1, 2, 4, 5, 6, 8, 9, or 16) (Zhang et al., 2006). Nevertheless, the *Fgf10* and *Fgfr2* null mice share the majority of defects within the orofacial area, with the exception of milder tooth and inner ear defects in *Fgf10* mutant mice (Kettunen et al., 2000; Ohuchi et al., 2000; Pirvola et al., 2000), and development of medial nasal glands, which are absent in *Fgfr2* null mutants but form normally in *Fgf10* null mice (May et al., 2016). The milder phenotypes in *Fgf10* mutants are mostly explained by compensation by FGF3 (Kettunen et al., 2000; Wright, 2003) or FGF7 (May et al., 2016). Under certain conditions, FGF10 can also likely bind to FGFR1.

Because of the perinatal lethality of *Fgf10* null mutants, some of the functions of FGF10 can be revealed only in conditional knock-outs. Even the conditional approach is complicated by the fact that many developmental events within the orofacial area overlap both in timing and also in expression of similar genes, so the choice of induction time and appropriate driver is challenging, e.g., to avoid simultaneous cleft palate formation. Other approaches such as genetic rescue by changing FGF10 dosage in particular mutants may be used and can bring valuable information, but these must be interpreted with caution, as can be seen for example in the case of the Apert syndrome model.

The impact of absence or malfunction of FGF10 is apparent not only from the animal model data but also from findings in human patients. Thus, the FGF10 pathway presents a potential pharmacological target for cure of rare diseases related to overactivated or downregulated FGFR2 signaling. Also, this knowledge lays the groundwork for potential medical treatment to harness the regenerative potential of gland tissues, after damage. A number of regenerative approaches are being developed and tested in animal models (Lombaert et al., 2011; Garg and Zhang, 2017; Emmerson et al., 2018). For example, healthy lacrimal epithelial cell progenitor cultures (ECPCs) were isolated and cultured in the presence of FGF10 to achieve budding and engraftment in injured lacrimal glands (Gromova et al., 2017). In theory, engraftment of such cells taken directly from cancer patients before radiotherapy could in the future serve as a source of tissue regeneration. In general, knowledge of the molecular cascades functioning during physiological development provides a base for regenerative approaches where FGF10 or its downstream targets can be provided to cultured tissues to be used for engraftment. In the future, perhaps FGF10 could be directly supplied *in situ*, which could help patients with tissue damage or patients with congenital diseases caused by aberrant FGF10 function.

## Author Contributions

MP and OK took the lead in writing the manuscript. MP, JP, and PM produced the samples and characterized them by micro-CT analysis. All authors provided critical feedback and revised the manuscript.

## Conflict of Interest Statement

The authors declare that the research was conducted in the absence of any commercial or financial relationships that could be construed as a potential conflict of interest.
