# Supplementary material for: Bones, Glands, Ears and More: The Multiple Roles of FGF10 in Craniofacial Development
Source: Front Genet. 2018 Nov 16;9:542. doi: 10.3389/fgene.2018.00542 (PMC6250787; doi:10.3389/fgene.2018.00542)
Supplement: Supplementary file 1 [file Data_Sheet_1.PDF]

Supplementary Table 1. Summary of the role of FGF10 in orofacial development. Source tissue = site of *Fgf10* expression; Receiving tissue = site of *Fgfr2 IIIb* expression; based on data from mouse or rat models. Unless stated differently, the mutant models are murine. N.D. = not determined; CVP = circumvallate papilla; E = epithelium; M = mesenchyme.

**Organ**	***Fgf10* null phenotype**	**Other models**	**Human defects associated with *Fgf10/Fgfr2***	**Source tissue**	**Receiving tissue**
Palate	Cleft palate (Rice et al. 2004)	Exogenous FGF10 rescues soft palate phenotype in *Dlx5* null mice (Sugii et al. 2017)	Cleft lip and/or palate (GWAS studies - Shi et al. 2009; Yu et al. 2017)	M of palatal shelves (Rice et al. 2004)	E of palatal shelves (Rice et al. 2004)
Eye lid	Open eye-lid (perinatally) (Tao et al. 2005)	N.D.	N.D.	M beneath nascent eye-lid (Tao et al. 2005)	Epidermal cells of nascent eye-lid (Tao et al. 2005)
Skull	No pronounced phenotype	Lower dose of *Fgf10* rescues Apert syndrome-like phenotype (Hajihosseini et al. 2009)	Apert syndrome (GOF mutations) (Anderson et al. 1998)	M of sutures; osteoprogenitors of frontal bone (Veistinen et al. 2009)	M of sutures (ectopic expression of *Fgfr2 IIIb* observed in mice hemizygous for *Fgfr2 IIIc;* Hajihosseini et al. 2001)
Tongue	Partial ankylosis; altered shape; diminished CVP; larger fungiform papillae (Rice et al. 2004; Petersen et al. 2011; Prochazkova et al. 2017)	*Fgf10* activation in *Tak1* deficient mice increases cell proliferation and height of tongue (Song et al. 2013)	N.D.	M of tongue (Rice et al. 2004)	E of tongue (Rice et al. 2004)
Inner ear	Agenesis of the posterior semicircular canal, minor pathologies in morphology of other structures (e.g. anterior and laterral canals, cochlea, hair cells) (Pauley et al. 2003)	More severe phenotype observed in *Fgf3/10* dKOs - failure of otic vesicle formation (Pirvola et al. 2000; Alvarez et al. 2003)	Hearing loss and cochlear dysplasia in LADD syndrome (LOF mutations) (Lemmerling et al. 1999; Milunsky et al. 2006)	E of otic placode, otic vesicle, otocyst, canal cristae, cochlea; sensory neurons (Pauley et al. 2003; Pirvola et al. 2000)	E of otic placode, otic vesicle, otocyst, cochlear duct, semicircular canals; mild expression of *Fgfr2 IIIb* observed in mesenchyme (Pirvola et al. 2000)
Teeth	Absence of cervical loop in incisor; slightly smaller molar tooth (Harada et al. 1999; Ohuchi et al. 2000)	*Fgfr2* null mutant shows agenesis of molar tooth; *Fgf10* loss is likely compensated by FGF3 in molar (Ohuchi et al. 2000, Kettunen et al. 2000)	Thin enamel and peg-shaped incisors in LADD syndrome patients (Milunsky et al. 2006)	Condensing M beneath developing tooth primordia (Kettunen et al. 2000)	E of developing tooth primordia (Kettunen et al. 1998)
Mandible	No pronounced phenotype	Overexpression of *Fgf10* in rat model causes elongation of mandible and enhanced chondrogenic differentiation (Terao et al. 2011)	Mandibular prognatism (Cruz et al. 2017)	M of the mandible (Terao et al. 2011)	E of anterior part of mandible (Terao et al. 2011)
Salivary gland	Agenesis (Ohuchi et al. 2000; Jaskoll et al. 2005)	*In vitro* explants need source of FGF10; Hypoplasia of salivary glands observed in $Fgf10^{+/-}$ mice; Mutations reducing FGF10 ability to bind heparan sulfates cause gland epithelium to branch rather than elongate (Rebustini and Hoffman 2009; Knosp et al. 2012; Jaskoll et al. 2005; May et al. 2015; Patel et al. 2007; Makarenkova et al. 2009)	ALSG, LADD syndrome (Entesarian et al. 2007; Scheckenbach et al. 2008; Seymen et al. 2017; Milunsky et al. 2006)	M adjacent to developing gland (Chatzeli et al. 2017)	E of budding gland (Makarenkova et al. 2000)
Lacrimal gland	Agenesis (Govindarajan et al. 2000; Makarenkova et al. 2000)	Development of lacrimal gland is abolished in *Hs2st/Hs6st* (heparan sulfates sulfotransferases) double mutants (Qu et al. 2011)		M adjacent to developing gland (Makarenkova et al. 2000)	E of budding gland (Makarenkova et al. 2000)
